# Toll-like receptors in systemic lupus erythematosus: potential for personalized treatment

**DOI:** 10.3389/fphar.2014.00265

**Published:** 2014-12-08

**Authors:** Teja Celhar, Anna-Marie Fairhurst

**Affiliations:** Singapore Immunology Network, Agency for Science, Technology and Research (A*STAR), Singapore, Singapore

**Keywords:** systemic lupus erythematosus, TLR7, TLR9, type I interferons, pharmacogenomics

## Abstract

Systemic lupus erythematosus (SLE) is a complex autoimmune disease characterized by the loss of tolerance to self-nuclear antigens. The symptoms of SLE, progression of pathology and the array of autoantibodies present in the serum differ significantly from patient to patient, which calls for a personalized approach to treatment. SLE is polygenic and strongly influenced by gender, ethnicity, and environmental factors. Data from genome-wide association studies suggests that polymorphisms in as many as 100 genes contribute to SLE susceptibility. Recent research has focused on genes associated with Toll-like receptors (TLRs), type I interferons, immune regulation pathways, and immune-complex clearance. TLR7 and TLR9 have been extensively studied using lupus-prone mouse models. In multiple systems overexpression of TLR7 drives disease progression but interestingly, a loss of TLR9 results in an almost identical phenotype. While TLR7 overexpression has been linked to human SLE, the possible role of TLR9 in human disease remains elusive. In the present review, we focus on TLR polymorphisms and TLR expression in SLE patients and discuss their potential as biomarkers for individualized treatment.

## INTRODUCTION

Systemic lupus erythematosus (SLE) is a chronic autoimmune disease with a variety of clinical manifestations that differ from patient to patient. The heterogeneity of the symptoms represents a challenge for both diagnosis and treatment. While the diagnostic criteria for SLE have evolved over the past decades (reviewed in Yu et al., 2014), the treatment has remained largely symptomatic using non-specific conventional therapies which include non-steroidal anti-inflammatory drugs (NSAIDs), glucocorticoids, hydroxychloroquine (HCQ), and immunosuppressants (Murphy et al., 2013). In addition to their modest therapeutic effect, these drugs have severe side effects leading to substantial morbidity and mortality, resulting in a substantial economic burden to many societies (Lau and Mak, 2009). In this short review, we briefly discuss novel therapeutic agents and emerging immunological targets. We will focus on the burgeoning data on Toll-like receptor (TLR) 7 and TLR9 in SLE and on the factors that could influence their expression. We propose altered expression of TLR7/9 as a biomarker for identification of a subset of SLE patients that might benefit from a targeted therapeutic approach.

### RECENT DEVELOPMENTS IN B CELL THERAPEUTICS

Recently, therapeutic avenues in SLE have pursued biologic drugs which either deplete B cells or reduce their activity. Belimumab (Benlysta^®^) is the only biological drug and the first therapeutic in 50 years to be approved by the Federal Drug Administration (FDA) for the treatment of lupus. Belimumab is a human monoclonal antibody (mAb) specific for B lymphocyte stimulator (BLyS) protein/B cell activating factor (BAFF; Hahn, 2011). Multicenter randomized controlled trials demonstrated a significant reduction in the Safety of Estrogen in Lupus Erythematosus National Assessment–SLE Disease Activity Index (SELENA–SLEDAI) score and risk of severe flares (Furie et al., 2011; Navarra et al., 2011). Recent results also suggest that belimumab may improve renal disease; however, further studies are needed to demonstrate a benefit in patients with severe active renal nephritis (Dooley et al., 2013). Based on the success of belimumab, other B cell targeting therapies are being assessed in clinical trials, including rituximab (anti-CD20), epratuzumab (anti-CD22), blisibimod and tabalumab (anti-BLyS), and atacicept [anti-BLyS/APRIL (A proliferation-inducing ligand); Kamal, 2014]. So far, the outcomes of B cell depletion with rituximab in clinical trials have been disappointing, partially due to poor trial design, problems with outcome measures and lack of long-term follow-up (Looney et al., 2010; Murphy et al., 2013; Kamal, 2014). In a randomized, double-blind, placebo-controlled Phase III trial rituximab successfully depleted B cells in lupus nephritis patients and decreased anti-dsDNA antibody levels, but failed to improve renal disease (Rovin et al., 2012). Since human prospective studies and mouse models have suggested a multi-step hypothesis to the development of SLE, it is perhaps unsurprising that reducing B cells has only moderate effects in end organ disease such as nephritis (Arbuckle et al., 2003; Fairhurst et al., 2006; Kanta and Mohan, 2009). Based on this hypothesis, the B cells have a critical role in autoantibody production due to initial loss of tolerance. However, an additional amplification step within the innate immune system is needed for the development of end organ disease.

**FIGURE 1:**
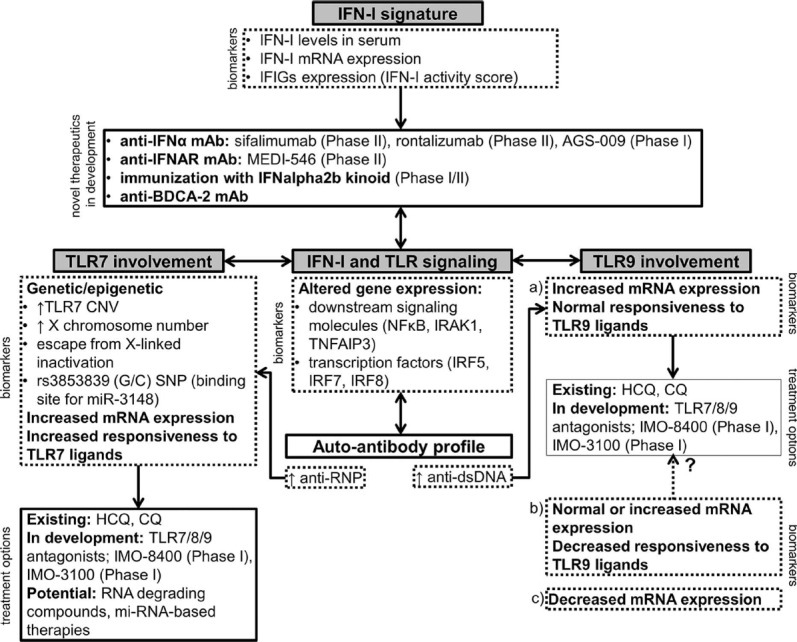
**A schematic representation of novel therapeutic approaches in SLE involving the IFN-I signature, TLR7 and TLR9.** The choice of the appropriate treatment option should ideally be based on the expression of relevant biomarkers in an individual patient. Drugs that are currently in clinical development are listed with the corresponding phase of the clinical trial (Clinicaltrials.gov, 2014). IFIGs, interferon-inducible genes; HCQ, hydroxychloroquine; CQ, chloroquine.

Accordingly, new therapeutic approaches which focus on targeting innate immune cells are being developed (Figure 1).

## TLR7 AS A TARGET FOR SLE THERAPY

### IMMUNOLOGICAL EVIDENCE FOR TLRs IN SLE

The type I-interferon (IFN-I) gene signature is one of the main immunological characteristics which was identified just over a decade ago by multiple groups (Baechler et al., 2003; Bennett et al., 2003; Crow, 2014). The increase in IFN-I-related genes was identified in the peripheral blood monocytes (PBMCs) from patients with SLE using gene expression profiling and has been identified in the majority of pediatric SLE patients and in the majority of adult SLE patients with active disease (Baechler et al., 2003; Bennett et al., 2003). Interestingly, the gene expression signature does not correlate with either elevated IFN-I levels in SLE serum, which is detectable only in a fraction of patients, or with elevated IFN-I mRNA (Blanco et al., 2001; Baechler et al., 2003). The reason for this discrepancy might be the low sensitivity of enzyme-linked immunosorbent assays (ELISAs) to detect serum IFN-I and the migration of IFN-I-producing cells to the tissues. However, there is also the possibility that the IFN-I signature is caused by another stimulus aside from IFN-I itself (Baechler et al., 2003; Bennett et al., 2003).

To overcome the limitations of serum IFN-I measurements “IFN-I activity score” assays were developed by several groups (Hua et al., 2006; Burgi Mde et al., 2012). These assays use serum or plasma of patients and the activity score strongly correlates with the titer of antinuclear autoantibodies (ANAs; Kirou et al., 2005; Hua et al., 2006; Niewold et al., 2007). High IFN-I activity score and the tendency to develop anti-ribonucleoprotein (RNP) and anti-dsDNA antibodies appear to be independent risk factors for SLE, since healthy first-degree relatives of SLE patients frequently display elevated IFN-I activity but no detectable ANAs (Niewold et al., 2007). The crucial connection between both traits is nucleic acid-sensing by endosomal TLRs, which evolved as sensors of foreign RNA and DNA (Blasius and Beutler, 2010; Kawai and Akira, 2010). In SLE, where self-nucleic acids are associated with autoantibodies in immune complexes (ICs), endosomal TLRs might become aberrantly activated in the absence of foreign molecules (reviewed in Celhar et al., 2012). Multiple *in vitro* studies in both mouse and human cells proved that RNA/DNA-containing ICs activate TLR9 and TLR7 through B cell receptor (BCR)-mediated internalization in B cells (Leadbetter et al., 2002; Lau et al., 2005) and through Fc gamma receptor (FcγR)-mediated internalization in dendritic cells (DCs; Boule et al., 2004), plasmacytoid DCs (pDCs; Bave et al., 2003; Barrat et al., 2005; Means et al., 2005; Vollmer et al., 2005; Lovgren et al., 2006), macrophages (Henault et al., 2012), and neutrophils (Garcia-Romo et al., 2011). Upon such activation, pDCs produce IFNα (Bave et al., 2003; Barrat et al., 2005; Means et al., 2005; Vollmer et al., 2005; Lovgren et al., 2006), conventional DCs produce cytokines (Boule et al., 2004) and neutrophils release neutrophil extracellular traps (NETs; Garcia-Romo et al., 2011). These findings highlight the central role of TLR7 and TLR9 in the induction and modulation of immune responses in SLE, and their association with the IFN-I signature (recently reviewed in Kono et al., 2013; Shrivastav and Niewold, 2013; Crow, 2014). Genome-wide association studies (GWAS) have provided supporting evidence by identifying SLE susceptibility variants in both TLR and IFN-I pathways (Rullo and Tsao, 2013).

### GENETIC EVIDENCE: POLYMORPHISMS IN TLR7-ASSOCIATED PATHWAYS

Overexpression of TLR7 causes severe lupus in multiple mouse models of SLE (reviewed in Celhar et al., 2012). Male BXSB lupus mice develop severe disease due to a translocation of a segment near the pseudoautosomal region of the X chromosome onto the Y chromosome, identified as the y-linked autoimmune accelerating (*yaa*) locus (Pisitkun et al., 2006; Subramanian et al., 2006). Among the duplicated genes, *TLR7* was identified as the major gene responsible for the development of severe disease (Deane et al., 2007; Fairhurst et al., 2008; Santiago-Raber et al., 2008). Genetically modified mouse models have proven to be an indispensable tool for the study of single gene modifications. However, correlations with the highly genetically variable human population are rarely straightforward. This is particularly true for polygenic disorders such as SLE, where multiple genes are associated with disease susceptibility (Rullo and Tsao, 2013; Armstrong et al., 2014). Genomic data suggests that common copy number variations (CNVs) similar to *yaa* are very rare in human SLE (Conrad et al., 2010; Park et al., 2010; Shen et al., 2010). Nevertheless, they may be an important risk factor childhood-onset SLE, as recently shown in the Mexican population (Garcia-Ortiz et al., 2010). Male patients with more than one copy and female patients with greater than two copies of TLR7 had a higher disease susceptibility and the TLR7 copy number correlated with TLR7 mRNA expression levels (Garcia-Ortiz et al., 2010). In addition to altered gene copy number, the gene dosage of TLR7 can be altered in individuals affected by aneuploidy. Indeed, SLE is more common in men affected by conditions with additional X chromosomes, such as Klinefelter’s syndrome (47,XXY; Ortiz-Neu and LeRoy, 1969; Dillon et al., 2012).

Overall, however, it is unlikely that a single gene, in this case TLR7, would have such a large impact. Consistent with this theory, recent investigations have identified polymorphisms in genes that are shared among TLR and IFN-I signaling pathways, including the transcription factors interferon regulatory factor (IRF) 5, IRF7, and IRF8 and components of the downstream NFκB pathway, such as interleukin-1 receptor-associated kinase 1 (IRAK1) and tumor necrosis factor, alpha-induced protein 3 (TNFAIP3; Rullo and Tsao, 2013). Therefore, polymorphisms in several genes could ultimately together lead to an excessive response to RNA sensing by TLR7. Additionally, variants in regulatory single nucleotide polymorphisms (SNPs) which bind microRNA (miRNA), X-chromosome linked modifications and induction of TLR7 expression by various infectious agents may be involved in subgroups of SLE patients.

### X-CHROMOSOME LINKED ALTERATIONS OF TLR7 EXPRESSION

Recent findings suggest that approximately 15% of genes on the X chromosome escape X-linked inactivation and are thus bi-allelicially expressed, ultimately increasing gene dosage (reviewed in Berletch et al., 2011). X-linked genes are particularly interesting in dissecting out causes of conditions that predominately affect women, including autoimmune diseases and some viral infections such as HIV and herpes simplex virus (HSV; Fish, 2008). Several studies have attributed these sexual dimorphisms to differential RNA-sensing by *TLR7*, since female immune cells produce higher levels of IFNα upon stimulation with TLR7 ligands or virus-derived RNA compared to male counterparts (Berghofer et al., 2006; Meier et al., 2009; Torcia et al., 2012). The underling mechanism for this sex bias is currently not clear. Berghofer et al. (2006) found equivalent *TLR7* mRNA expression in pDCs and B cells from healthy males and females with no evidence of escaping X-linked inactivation or involvement of estrogen receptor signaling. However, these findings do not exclude escape from X-linked inactivation in SLE patients.

### EPIGENETIC FACTORS AND microRNA BINDING

It has now been established that miRNAs can act to fine tune TLR signaling by targeting expression itself or by modulating adaptor molecules, downstream regulators and cytokines (reviewed in O’Neill et al., 2011). Variants in the 3′ untranslated region (UTR) regions may alter miRNAs binding and ultimately TLR7 expression and/or responsiveness. A large multi-centered and multi-ethnic study identified such SNP in the 3′ UTR region of TLR7 (rs3853839 (G/C)) as a risk factor for SLE (Shen et al., 2010; Deng et al., 2013). The G-allele carriers have increased *TLR7* transcripts and are more likely to have anti-RNA associated autoantibodies than C-allele carriers (Shen et al., 2010). The non-risk C allele bears a binding site of microRNA-3148 (miR-3148), which confers faster degradation of the transcript and thus lower levels of *TLR7* gene product (Deng et al., 2013).

### INDUCTION OF TLR7 EXPRESSION

Several studies have reported the induction of TLR7 transcription in immune cells (B cells, eosinophils, monocytes, macrophages, pDCs) following stimulation with various inflammatory agents, including bacteria (Zarember and Godowski, 2002; Bourke et al., 2003; Miettinen et al., 2008), viruses (Miettinen et al., 2001) CpG (Hornung et al., 2002; Bourke et al., 2003), IFNα (Miettinen et al., 2001; Sirén et al., 2005), and IFNγ (Miettinen et al., 2001; Nagase et al., 2003). This increase in TLR7 during infection may be part of a positive feedback mechanism to increase IFNα, which is essential for a fast and robust anti-microbial response (Rönnblom et al., 2006). It is possible that this pathway is deregulated in SLE patients and explains why many features of SLE resemble a chronic viral infection in the absence of a detectable virus (Crow, 2014). Indeed, upregulated TLR7 and TLR9 mRNA expression have been reported in PBMCs from SLE patients and levels correlate with the expression of IFNα (Komatsuda et al., 2008; Lyn-Cook et al., 2014). Upregulation of TLR7, but not other TLRs, has also been observed when healthy neutrophils were cultured with sera from SLE patients with active disease (Garcia-Romo et al., 2011). This may be due to IFNα present in patient’s sera, since pre-treatment with purified IFNα resulted in a similar response of increased TLR7. Furthermore, IFNα increased susceptibility to anti-RNP antibody-induced NETosis (Garcia-Romo et al., 2011). Consistent with these findings, monocyte-derived macrophages produce IFNα upon TLR7/8 ligand stimulation only after pre-treatment with IFN*α* and subsequent induction of TLR7 (Sirén et al., 2005). TLR7 may also be induced by serum-derived ICs containing TLR7 ligands. A recent study by Chauhan et al. (2013) showed that TLR7 was preferentially increased in SLE patients with antibodies against RNA-associated antigens, while TLR9 induction correlated with anti-dsDNA antibody titers. Moreover, flu and synthetic TLR7 ligands have the capacity to induce early IFN-inducible genes in pDCs independently of IFN-α production (Di Domizio et al., 2009). Overall, accumulating data suggest that persistently higher levels of TLR7 may lead to an acquired responsiveness in cells which are normally unresponsive to TLR7 ligands. This is supported by the observation that pDCs are responsible for the majority IFN*α* produced by healthy PBMCs, but account only for 57% of IFN*α* produced by PBMCs from SLE patients (Blanco et al., 2001).

## TLR9 AS A TARGET FOR THERAPY FOR SLE

While TLR7 hyper-responsiveness in human SLE is consistent with the TLR7-associated nephritis shown by mouse models, the role of TLR9 remains controversial. Multiple mouse studies have shown the importance of TLR9 expression in B cells for the generation of anti-dsDNA, anti-chromatin, and anti-nucleosome autoantibodies (Christensen et al., 2005, 2006; Lartigue et al., 2006; Yu et al., 2006; Nickerson et al., 2010). However, the deletion of TLR9 in these lupus-prone models did not lead to amelioration, but rather to exacerbation of disease, suggesting a protective/regulatory role of TLR9 in cells other than B cells. Nonetheless, the manifestation of the disease in TLR9-deficient mice was dependent on TLR7 expression (Nickerson et al., 2010).

Increases in *TLR9* expression have been shown in PBMCs from SLE patients and levels usually correlate with *IFNα* expression and anti-dsDNA antibodies (Komatsuda et al., 2008; Mu et al., 2012; Chauhan et al., 2013; Lyn-Cook et al., 2014). Additionally, B cells and monocytes from patients with active disease express higher TLR9 levels compared to patients with inactive disease (Papadimitraki et al., 2006; Nakano et al., 2008). An increase in the frequency of TLR9-expressing B cells, but not monocytes, correlated with anti-dsDNA antibodies (Papadimitraki et al., 2006). These results suggest that, similarly to murine studies, increased TLR9 expression in B cells may be involved in autoantibody production, especially anti-dsDNA

However, paradoxically, the increased expression of TLR9 does not lead to increased responsiveness to TLR9 ligands. Despite an increase in TLR9 on B cells and BDCA3^+^ DCs from patients with severe disease, cells were less activated and hyporesponsive to TLR9 stimulation (Zorro et al., 2009). Similarly, despite an increased TLR9 expression in SLE PBMCs, TLR9-induced IFN-α production was markedly reduced (Kwok et al., 2008). This desensitization has been directly attributed to HCQ treatment (Kwok et al., 2008; Sacre et al., 2012). However, in patients not taking HCQ, alterations in downstream TLR9-signaling or induction of negative regulators may be involved (Kwok et al., 2008; Zorro et al., 2009).

Limited data is available on the effect of decreased TLR9 expression in human SLE. A recent study conducted in a cohort of Danish SLE patients found significantly decreased *TLR9* mRNA expression in lupus PBMCs, which did not correlate with dsDNA antibody titers (Laska et al., 2014). It would be interesting to further identify the main cell type within the PBMCs responsible for the downregulation and assess the responsiveness to TLR9 ligands in this cohort.

Overall, further research is required to establish the role of TLR9 in mediating the progression of benign autoimmunity to severe disease.

### TLR9 POLYMORPHISMS

Several TLR9 polymorphisms have been associated with SLE, particularly in patients of Asian or European ancestry (Tao et al., 2007; Xu et al., 2009; Lu et al., 2011; dos Santos et al., 2012; Huang et al., 2012; Piotrowski et al., 2013; Laska et al., 2014). However, these results were not confirmed by other studies or by a recent meta-analysis (Hur et al., 2005; Ng et al., 2005; De Jager et al., 2006; Yang et al., 2012). Similarly, as mentioned earlier for TLR7, polymorphisms in a single gene are not very likely to recapitulate a polygenic disease like SLE. Additional genetic variants in TLR9-downstream signaling pathways, regulators of TLR signaling and epigenetic modifications should also be considered and need further examination.

## DEVELOPMENT OF NOVEL THERAPEUTICS FOR SLE

### NOVEL THERAPIES DIRECTED TOWARD IFNα

IFNα signaling may be suppressed by several strategies: direct neutralization by an anti-IFNα mAb, suppression of the IFN-I signature using an anti-IFNα receptor (IFNAR) antibody, or immunization to achieve endogenous anti-IFNα production (reviewed in Crow, 2014). Three anti-IFN-α mAbs, sifalimumab, AGS-009, and rontalizumab, have achieved safety and dose-dependent reduction of IFN-I signature in Phase I SLE trials (Yao et al., 2009; Merrill et al., 2011; Petri et al., 2013; Stohl, 2013). Furthermore, a Phase II trial has shown that rontalizumab decreased the incidence of flares and reduced prednisone dosage in a subgroup of patients who had a low IFN-I signature at baseline (Stohl, 2013).

Targeting the IFNAR with MEDI-546, a fully human mAb against huIFNAR, achieved success in Phase I trials for systemic sclerosis (SSc; Wang et al., 2013). Pharmacogenomics and translational simulations were used to build a model to predict responses to MEDI-546 in SLE patients and facilitated the progression of this candidate molecule to a Phase II trial (Wang et al., 2013).

Immunization with human (hu) IFNalpha2b kinoid (IFN-K) generates endogenous polyclonal antibodies directed against all 13 subtypes of huIFNα (Mathian et al., 2011). The results from the first clinical study show that IFN-K is well tolerated and significantly reduces the IFN-I signature in IFN signature-positive patients (Lauwerys et al., 2013).

An additional approach is the blockage of BDCA-2 expressed on the surface of pDCs. Anti-BDCA-2 mAb successfully inhibited IFN-α production from healthy donors and SLE patients (Dzionek et al., 2001; Blomberg et al., 2003). A patent application has recently been submitted for an anti-BDCA-2 antibody in the context of preventing or treating pathological conditions involving pDC activation (De and Fournier, 2012).

### TLR INHIBITION IN SLE

Several compounds that bind endosomal TLR7 and/or TLR9 activation have been developed with the goal to inhibit IFNα production and activation of autoimmune B cells (Barrat and Coffman, 2008; Kandimalla et al., 2013). This approach successfully ameliorated disease in lupus-prone mice but it needs further clinical evaluation (Barrat et al., 2007). IMO-3100, a TLR7/9 antagonist, and IMO-8400, a TLR7/8/9 antagonist, suppressed inflammation in a mouse model of psoriasis (Suarez-Farinas et al., 2013). Both compounds were well tolerated by healthy adults and are now undergoing Phase 2 trials in psoriasis patients (Clinicaltrials.gov, 2014). It is important to note that the therapeutic value of the standard SLE care drugs HCQ and chloroquine have been attributed to indirect inhibition of TLR7 and TLR9, through their inhibitory action on endosomal acidification which is necessary for TLR7/9 activation (Sacre et al., 2012).

Finally, miRNA-based therapies and therapeutics design to degrade RNA may also represent potential treatment options in specific groups of SLE patients (Deng et al., 2013; Sun et al., 2013).

## CONCLUSION

Advances in the understanding of the innate immune system have revolutionized the development of novel SLE therapies. Owing to the heterogeneous nature of the clinical manifestations, much thought is being given to a stratification of lupus patients based on clinical symptoms and immunological dysfunctions, targeting disease development in a personalized medicine approach. This is particularly important for the design of clinical trials, where the correct recruitment and sub-grouping of patients may directly impact the outcome measures. Pharmacogenomic and pharmacogenetic studies will ultimately be essential to identify patients that will most likely benefit from a specific treatment.

### Conflict of Interest Statement

The authors declare that the research was conducted in the absence of any commercial or financial relationships that could be construed as a potential conflict of interest.
